# Edward Ratcliffe Holborow

**Published:** 1923-10

**Authors:** 


### EDWARD RATCLIFFE HOLBOROW, M.B., B.S. Lond., F.R.C.S. Ed.

On August 18th in a very rough sea at Weston-super-Mare two ladies bathing were in grave danger, and Dr. Holborow was drowned in attempting to rescue them. He had never fully recovered health and strength undermined in air war service; yet unequal to the strain and stress involved by his self-sacrificing bravery, it was characteristic of the man to respond to the call and ignore the odds against him, which he was so physically unfitted to combat.

Holborow's family for generations have belonged to the Badminton country. He was educated at the University of Bristol, where he won the Lady Haberfield Prize, and graduated as M.B. and B.S. at the London University in 1909, taking the F.R.C.S. Ed. in 1917.

Holborow was House Surgeon at Somerset Hospital, Cape Town, and also at the Pretoria Hospital, Transvaal.

On the outbreak of war he joined the R.A.M.C. and served in Gallipoli, and after the evacuation thereof on the western front in France. He was subsequently Surgical Specialist at the Fovant Military Hospital on Salisbury Plain.

On demobilisation he settled in practice at Weston-super-Mare, where he was appointed Poor Law Medical Officer.

Holborow's quiet charm of manner and amiability endeared him to all his colleagues and patients of all classes, and made many friends. The admiration of his fellow-townsmen for his gallantry was marked by a public funeral, and the widespread sympathy for his widow and family of two very young children.

